# PET/MRI for Staging the Axilla in Breast Cancer: Current Evidence and the Rationale for SNB vs. PET/MRI Trials

**DOI:** 10.3390/cancers13143571

**Published:** 2021-07-16

**Authors:** Rosa Di Micco, Letizia Santurro, Maria Luisa Gasparri, Veronica Zuber, Giovanni Cisternino, Sara Baleri, Manuela Morgante, Nicole Rotmensz, Carla Canevari, Francesca Gallivanone, Paola Scifo, Annarita Savi, Patrizia Magnani, Ilaria Neri, Nadia Ferjani, Elena Venturini, Claudio Losio, Isabella Sassi, Giampaolo Bianchini, Pietro Panizza, Luigi Gianolli, Oreste Davide Gentilini

**Affiliations:** 1 Breast Surgery Unit, San Raffaele University and Research Hospital, Via Olgettina 60, 20132 Milan, Italy; santurro.letizia@hsr.it (L.S.); zuber.veronica@hsr.it (V.Z.); cisternino.giovanni@hsr.it (G.C.); baleri.sara@hsr.it (S.B.); morgante.manuela@hsr.it (M.M.); rotmensz.nicole@hsr.it (N.R.); gentilini.oreste@hsr.it (O.D.G.); 2 Department of Gynecology and Obstetrics, Ente Ospedaliero Cantonale, Ospedale Regionale di Lugano, Via Tesserete 46, 6900 Lugano, Switzerland; marialuisa.gasparri@eoc.ch; 3 Faculty of Biomedicine, Università Della Svizzera Italiana, Via Giuseppe Buffi 13, 6900 Lugano, Switzerland; 4 Department of Experimental Diagnostic and Specialty Medicine (DIMES), IRCCS Policlinico S.Orsola Hospital, Università di Bologna, Via Giuseppe Massarenti 9, 40138 Bologna, Italy; 5 Nuclear Medicine Department, San Raffaele University and Research Hospital, Via Olgettina 60, 20132 Milan, Italy; canevari.carla@hsr.it (C.C.); scifo.paola@hsr.it (P.S.); savi.annarita@hsr.it (A.S.); magnani.patrizia@hsr.it (P.M.); neri.ilaria@hsr.it (I.N.); ferjani.nadia@hsr.it (N.F.); gianolli.luigi@hsr.it (L.G.); 6 Institute of Molecular Bioimaging and Physiology, National Research Council (IBFM-CNR), Via Fratelli Cervi, 20090 Milan, Italy; 7 Breast Radiology Unit, San Raffaele University and Research Hospital, Via Olgettina 60, 20132 Milan, Italy; venturini.elena@hsr.it (E.V.); losio.claudio@hsr.it (C.L.); panizza.pietro@hsr.it (P.P.); 8 Pathology Unit, San Raffaele University and Research Hospital, Via Olgettina 60, 20132 Milan, Italy; sassi.isabella@hsr.it; 9 Breast Cancer Group, Department of Medical Oncology, San Raffaele University and Research Hospital, Via Olgettina 60, 20132 Milan, Italy; bianchini.giampaolo@hsr.it

**Keywords:** breast cancer, PET/MRI, sentinel node biopsy

## Abstract

**Simple Summary:**

PET/MRI is a relatively new, hybrid imaging tool that allows practitioners to obtain both a local and systemic staging in breast cancer patients in a single exam. To date, the available evidence is not sufficient to determine the role of PET/MRI in breast cancer management. The aims of this paper are to provide an overview of the current literature on PET/MRI in breast cancer, and to illustrate two ongoing trials aimed at defining the eventual role of PET/MRI in axillary staging in two different settings: patients with early breast cancer and patients with positive axillary nodes that are candidates for primary systemic therapy. In both cases, findings from PET/MRI will be compared with the final pathology and could be helpful to better tailor axillary surgery in the future.

**Abstract:**

Axillary surgery in breast cancer (BC) is no longer a therapeutic procedure but has become a purely staging procedure. The progressive improvement in imaging techniques has paved the way to the hypothesis that prognostic information on nodal status deriving from surgery could be obtained with an accurate diagnostic exam. Positron emission tomography/magnetic resonance imaging (PET/MRI) is a relatively new imaging tool and its role in breast cancer patients is still under investigation. We reviewed the available literature on PET/MRI in BC patients. This overview showed that PET/MRI yields a high diagnostic performance for the primary tumor and distant lesions of liver, brain and bone. In particular, the results of PET/MRI in staging the axilla are promising. This provided the rationale for two prospective comparative trials between axillary surgery and PET/MRI that could lead to a further de-escalation of surgical treatment of BC. • SNB vs. PET/MRI 1 trial compares PET/MRI and axillary surgery in staging the axilla of BC patients undergoing primary systemic therapy (PST). • SNB vs. PET/MRI 2 trial compares PET/MRI and sentinel node biopsy (SNB) in staging the axilla of early BC patients who are candidates for upfront surgery. Finally, these ongoing studies will help clarify the role of PET/MRI in BC and establish whether it represents a useful diagnostic tool that could guide, or ideally replace, axillary surgery in the future.

## 1. Introduction

Over two million new cases of breast cancer (BC) are diagnosed every year worldwide. Over time, the supremacy of a radical surgery has been replaced by a less invasive surgery. In fact, breast conserving surgery (BCS) and sentinel node biopsy (SNB) are performed in more than 70% of BC patients [1,2]. This de-escalation of breast and axillary surgery has gone hand in hand with the improvement in imaging techniques, as well as systemic therapy and radiotherapy that also contribute to local control [3,4].

Modern diagnostic imaging tools provide an accurate local and systemic staging in order to plan the primary treatment and to tailor the best surgical procedure. Whilst mammography, ultrasound (US) and magnetic resonance imaging (MRI) represent an excellent option to stage the T, staging the axilla with imaging is still challenging. To date, several studies have demonstrated the limitations of axillary ultrasound (Ax-US); these include the fact that it is an operator-dependent technique, its sensitivity ranges from 23% to 80% and also, it is unable to estimate the true axillary tumor burden [5,6]. Similarly, other tools such as standard breast MRI [7], Positron Emission Mammography (PEM) [8], PET/CT [9] are not accurate enough to predict axillary stage. On the one hand, two large meta-analyses have shown that Ax-US and selective needle biopsy correctly identifies around 50–55% of node-positive patients [6,10]. On the other hand, when considering the tumor burden, having abnormal nodes on Ax-US, mammogram and MRI often equates to having only 1–2 positive sentinel nodes that do not always change surgical plans [7,11,12]. However, the accuracy is not excellent and even when Ax-US identifies fewer than two abnormal nodes, patients are still more likely to have more than three positive nodes [13].

At first, axillary surgery had a curative intent and axillary dissection (AD) was always indicated; thereafter, SNB replaced AD and axillary surgery was more intended as a way to derive information on axillary status and plan adjuvant treatments. In fact, historical trials demonstrated no survival advantage in performing AD, and showed that it could cause more complications, long-term morbidities and, indeed, a worse quality of life [14,15,16,17]. Over time, AD has been progressively abandoned: IBCSG 23-01, ACOSOG Z0011 and AMAROS trials showed no survival advantage in completing AD in cT1-2 tumors with a positive sentinel node [16,17,18]. In parallel, primary systemic therapy (PST) has started to downstage positive axillae where AD was initially indicated and de-escalate final axillary surgery [19].

Considering this gradual switch in the role of axillary surgery from a therapeutic to a staging procedure, the role of imaging has strongly increased. Ideally, in the future, imaging might even replace surgery in the axillary staging of BC patients [20,21], while still providing reliable information to guide medical treatments. Today, systemic therapy is increasingly based on tumor biology rather than on nodal status, and gene expression signatures can also help decide on adjuvant treatment [22]. In this context, achievement of the most accurate preoperative imaging assessment of the axilla, in order to decide the most appropriate treatment for each patient, is an unmet need.

## 2. What Is the Role of PET/MRI in Breast Cancer?

PET/MRI is a relatively new imaging tool, and its field of application is still being studied. It was introduced in 2011 in the USA and UE, offering the potential to combine the specificity obtained by the functional imaging of PET with the superior sensitivity of MRI, and provide relevant information of higher diagnostic accuracy [23]. In particular, the fully integrated PET/MRI system provides a simultaneous imaging acquisition [24].

Since its introduction, PET/MRI was conceived as a new imaging tool to compare with the pre-existing PET/CT, with the idea that it could have an added value and outperform the previous imaging available in some selected organs [25]. In fact, the first studies on PET/MRI show its application in systemic staging in cancer patients, in both pre-surgical and follow-up settings [26,27,28,29]. Once the staging power of PET/MRI was demonstrated to be similar, or even superior, to PET/CT, researchers focused their attention on studying how the PET/MRI performs in single organs. In the last decade, the breast has become one of the most interesting fields of application, not only for staging purposes but also to characterize benign versus malignant lesions, assess the response to systemic therapy in neoadjuvant settings, and even to predict the prognosis [30,31,32]. Generally, the integrated whole-body PET/MRI was demonstrated to be feasible in a clinical setting, providing high quality, and a short examination time [27]. The reliability of PET/MRI seemed to be comparable [26,27,29], or even superior [25], to PET/CT in systemic cancer staging.

As regards BC, the application of PET/MRI was studied in four different settings: for preoperative staging at diagnosis, for follow-up staging, to predict the prognosis and the response to therapy (Table 1). In particular, Kirchner et al. [30] demonstrated the necessity and superiority of a two-step 18F-FDG-PET/MRI imaging algorithm, comprising dedicated prone breast imaging and supine whole-body imaging, when compared with the one-step algorithm for both staging. This now represents the standard protocol for BC staging [33,34,35]. Considering the BC lesions, the axillary nodes and the metastatic lesions, PET/MRI showed an equivalent performance in terms of qualitative lesion detection to PET/CT, but it had a superior sensitivity and lower specificity in the lesion-per-lesion analysis, especially for bone and liver metastases [36,37,38]. Additionally, Melsaether et al. [39] highlighted a lower sensitivity for pulmonary metastases, thereby confirming previous studies [28], as also mentioned above. Furthermore, three studies [40,41,42] compared the performance of the combined PET/MRI with each single exam, PET and/or MR alone, respectively. All studies found that MRI alone has the highest sensitivity for primary tumors and both MR and PET/MRI are highly specific for nodal metastases.

On the one hand, the advantages of this hybrid diagnostic tool are a lower radiation dose when compared to PET/CT, better inter-observer agreement, a one-stage exam and more accurate detection of brain, bone and liver metastases. On the other hand, PET/MRI is still an expensive and time-consuming imaging method, which is not available everywhere; despite the attractiveness of performing a single exam when both PET and MR imaging are indicated, PET/MRI also exhibits other limitations (i.e., long duration, MR truncation, PET/MRI misregistration, etc.) [43].

To conclude, the role of PET/MRI in the BC setting is not yet well defined, although it shows good accuracy in BC local and systemic staging and could be considered in both monitoring and predicting the response to PST. However, the heterogeneity of the studies reported and the variability of the PET/MRI approach limit the comparison and the summation of data. Hence, current evidence is not sufficient to derive standard indications; ongoing and future research on PET/MRI could help clarify its role and establish whether it may represent a useful diagnostic and prognostic tool, or if it needs to be replaced or integrated with other conventional diagnostic tools.

## 3. PET/MRI in Axillary Staging: Current Evidence

Several studies have investigated the power of PET/MRI in staging the axilla; the results are encouraging but preliminary, due to the small sample size and inhomogeneous study population and design (Table 2). Most of these studies investigated the axillary status at diagnosis, whilst the minor part considered the PET/MRI performed during the follow-up phase to discover eventual recurrences. Although an additional PET/MRI imaging does not seem to change plans after MRI imaging, Taneja et al. [41] found a higher diagnostic confidence of PET/MRI in classifying a node as positive when compared to PET or MRI alone. However, this was a pilot study, which recruited 36 patients, and only 15 had positive axilla at diagnosis, so the final results should be confirmed on a larger sample.

Regarding the N-stage, Melsaether et al. [39] showed a sensitivity from 88% to 100%, whilst Grueneisen et al. [42] reported a specificity of 94% and sensitivity of 78% for the detection of lymph node metastasis, which was no different from the findings for PET/CT and MRI alone. Hence, the conventional staging procedures and invasive techniques for staging the axilla are still recommended. Interestingly, Goorts et al. [45] studied the added value of PET/MRI imaging in patients undergoing PST and compared these results to conventional imaging (i.e., mammography, US, MRI). They found no added value of PET/MRI in tumor staging, while in 10% of patients, nodal or distant metastases were discovered and, in another 10%, PET/MRI imaging confirmed the malignancy of a suspicious lesion on MRI, making eventual PET/CT imaging and tissue sampling redundant. Additionally, this study highlighted an extra asset in the usability of this imaging modality, i.e., evaluating the exact number of positive axillary and extra-axillary nodes in order to choose the best treatment option (i.e., PST versus upfront surgery, radiation fields), while conventional staging methods could have missed this information. More recently, van Nijnatten et al. [44] experimented with a dedicated axillary 18F-FDG hybrid PET/MRI in 12 patients with BC that allowed for better delineation of the lymph nodes. This additional imaging tool resulted in a change in nodal status in 40–75% of patients when compared to US or MRI, and in 22% when compared to PET/CT, thereby adding value to PET/MRI in nodal staging. Despite these promising results, as the authors underline in the limitations, no final pathology was available in this study to confirm lymph-node positivity, and PET/CT was obtained as an unenhanced low-dose exam, which certainly could have affected the results.

## 4. The Rationale of SNB vs. PET/MRI Trials

After the paradigm shift that confined the role of axillary surgery in BC treatment to a purely staging procedure, the idea of replacing surgery with a less invasive technique was conceived [22]. Ongoing trials are evaluating the role of preoperative Ax-US in staging the axilla, but this remains an operator-dependent imaging tool [20,21,73,74]. The advent of an integrated PET/MRI system with an accurate spatial and temporal co-registration of PET and MRI data, which has provided the best of the two imaging techniques, could potentially offer a high level of diagnostic accuracy. Ideally, axillary imaging should help to differentiate between node-negative and node-positive BC patients and, among these, patients with a low and high axillary tumor burden, thereby allowing the surgical oncologist to decide on the best primary treatment.

To date, no studies with appropriate sample size and dedicated design have highlighted the potential relevance of PET/MRI in axillary staging in BC patients [41,45,57]. Furthermore, most previous studies focused on locally advanced BC, with limited data on early- and post-PST BC, in which tumor burden is lower and axillary imaging is even more complex. Table 3 reports ongoing studies evaluating the performance of PET/MRI in staging the axilla, and using two different rationales: three studies aim to compare the results with surgery, whilst another three trials focus on the comparison with standard imaging. On the one hand, the application of PET/MRI in BC treatment is intended to guide surgery in early- or post-PST BC settings, so the results from the first three studies could be valuable for the surgical oncologist (ClinicalTrials.gov ID: NCT04826211, NCT04829643, NCT03374826). On the other hand, the results from the last three trials could suggest adding PET/MRI to the pathway of BC treatment in place of other imaging tools. In particular, the two American studies aim to compare PET/MRI with MRI alone in newly diagnosed BC, thereby focusing on the performance of PET/MRI on the breast and axilla (ClinicalTrials.gov ID: NCT03510988); and PET/MRI with PET/CT in patients performing staging exams at diagnosis or on follow-up, thereby evaluating the impact of PET/MRI on whole-body staging (ClinicalTrials.gov ID: NCT01672021). Similarly, the last German trial will provide data on the additional value of PET/MRI at diagnosis, compared with standard imaging in the context of BC at high risk of metastasis (German Clinical Trials Register, DRKS register number: DRKS00005410). In a few years, the results from these trials could clarify whether PET/MRI is useful in BC patients to evaluate breast and lymph nodes or other organs, or all of these at the same time.

## 5. Italian Experience on SNB vs. PET/MRI Trials: Two Comparative Studies between PET/MRI and Axillary Surgery

The Italian SNB vs. PET/MRI trials were initiated in the Breast Surgery Unit of San Raffaele University and Research Hospital in Milan where a hybrid SIGNATM PET/MRI (General Electric Healthcare, Waukesha, WI, USA) is available.

The study hypothesis was that hybrid PET/MRI might be a non-invasive, one-stage, operator-independent imaging modality that could accurately define nodal status in BC patients. PET/MRI could eventually help select the proper type of surgical approach and might eventually lead to the omission of axillary surgery in the future.

These trials are two prospective comparative single-center studies carried out in two different settings:

- SNB vs. PET/MRI 1 trial (ClinicalTrials.gov Identifier: NCT04826211) evaluates axillary staging in nodal-positive BC patients receiving PST;
- SNB vs. PET/MRI 2 trial (ClinicalTrials.gov Identifier: NCT04829643) evaluates axillary staging in early BC patients undergoing upfront surgery.

Although they used diverse study populations, the two studies share common outcomes:

- The primary outcome was to compare the staging power between PET/MRI vs. SNB (or AD) in detecting axillary lymph node macro-metastases (>2 mm), evaluating the concordance rate between the two tools, which would indicate PET/MRI accuracy;
- The secondary outcome was to compare PET/MRI and Ax-US, evaluating the concordance rate between the two exams;
- The tertiary outcome was to investigate eventual associations between PET/MRI morphological or functional parameters and tumor prognostic features.

### 5.1. SNB vs. PET/MRI 1

**Figure 1:**
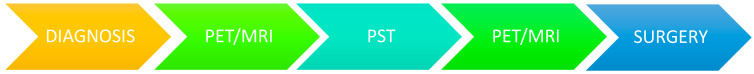
SNB vs. PET/MRI 1 flow-chart. (PST=Primary Sistemic Therapy).

A consecutive cohort of 110 patients with BC of any size and positive axillary nodes at diagnosis who are candidates for PST will be recruited from October 2019 (Table 4). Eligible patients are selected by the surgical or medical oncologist at the first consultation. After signing the informed consent, they will undergo routine diagnostic exams, including Ax-US. Additionally, PET/MRI is performed as a staging imaging tool before starting PST, and after PST before planning surgery together with a new Ax-US. Surgery is planned according to the final staging and nodal involvement at diagnosis, which is BCS or mastectomy for the breast, and SNB or AD for the axilla (Figure 1).

The study lasts five years. To date, 40 patients have already been recruited.

### 5.2. SNB vs. PET/MRI 2

**Figure 2:**
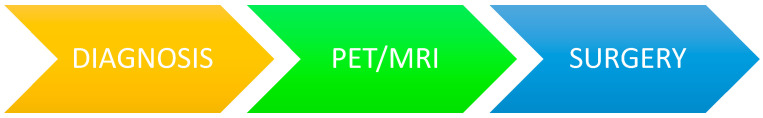
SNB vs. PET/MRI 2 flow-chart.

A consecutive cohort of 247 patients with early BC and without overt nodal involvement and who are candidates for surgery as primary treatment and SNB have been recruited since June 2020. Eligible patients are selected by the surgical oncologist at the first consultation. After signing the informed consent, they will undergo routine diagnostic exams, including Ax-US. Additionally, PET/MRI is performed as a staging imaging tool before surgery. Surgery could be BCS or mastectomy for the breast and SNB for the axilla (Figure 2).

The study lasts three years. To date, 40 patients have already been recruited.

## 6. Conclusions

The modern battle for the breast surgical oncologist aims to achieve the least invasive but effective treatment and eventually find an imaging tool that is able to predict pathological results and spare women from future axillary surgery.

To date, the current evidence does not permit the avoidance of surgery, but PET/MRI might offer patients a one-stop-shop solution for local and systemic staging, and guide the surgical oncologist to de-escalate axillary surgery in selected patients. Results from prospective trials on PET/MRI are anticipated in the next five years and should help decide the potential applications of this cutting-edge imaging tool in BC treatment.

## Figures and Tables

**Table 1:** Previous studies on PET/MRI in breast cancer patients divided according to the main objective of the exam into four groups: staging, follow-up, prognosis and response to therapy. (Nr.BC/Tot pts.: Number of breast cancer patients/total patients; NA: not available; WB: whole-body PET/MRI; B: breast PET/MRI).

Category Group	Reference	Total Number of Patients Nr. BC/tot. pts. (%)	Study Design	Patient Position	Type of Acquisition
**STAGING**	Catalano, O.A., 2013 [25] Huellner, M.W., 2014 [26] Drzezga, A., 2012 [27] Appenzeller, P., 2013 [28] Wiesmuller, M., 2013 [29] Kirchner, J., 2018 [30] Botsikas, D., 2019 [32] Pace, L., 2014 [36] Kong, E., 2014 [33] Melsaether, A.N., 2016 [39] Van Nijnatten, T.J., 2018 [44] Taneja, S., 2014 [41] Grueneisen, J., 2015 [42] Botsikas, D., 2016 [40] Catalano, O.A., 2017 [37] Goorts B., 2017 [45] Kirchner, J., 2020 [34] Bruckmann, N.M., 2020 [35] Bruckmann, N.M., 2021 [38]	35/134 (26.1%) 5/106 (4.8%) 3/32 (9.4%) 7/63 (11.1%) 3/46 (6.5%) 38/38 (100%) 80/80 (100%) 36/36 (100%) 42/42 (100%) 51/51 (100%) 12/12 (100%) 36/36 (100%) 49/49 (100%) 58/58 (100%) 51/51 (100%) 40/40 (100%) 56/56 (100%) 104/104 (100%) 154/154 (100%)	retrospective prospective prospective prospective prospective prospective prospective prospective prospective prospective prospective retrospective prospective retrospective retrospective prospective prospective prospective prospective	supine supine supine supine supine supine WB, prone B supine WB, prone B supine supine WB, prone B supine prone supine WB, prone B prone supine WB, prone B NA prone supine WB, prone B supine WB, prone B supine	simultaneous sequential simultaneous sequential simultaneous simultaneous sequential simultaneous simultaneous simultaneous simultaneous simultaneous simultaneous sequential simultaneous simultaneous simultaneous simultaneous simultaneous
**FOLLOW-UP**	Grueneisen, J., 2017 [31] Sawicki, L.M., 2016 [46] Pujara, A.C., 2016 [47] Beiderwellen, K., 2013 [48] Chandarana, H., 2013 [49] Rauscher, I., 2014 [50] Catalano, O.A., 2015 [51] Raad, R.A., 2016 [52] Ishii S., 2016 [53] Kirchner, J., 2017 [54] Sonni, I., 2019 [55]	36/36 (100%) 21/21 (100%) 35/35 (100%) 10/70 (14%) 10/32 (31.2%) 4/40 (10%) 109/109 (100%) 15/208 (7.2%) 33/123 (26.8%) 2/41 (5%) 23/74 (31%)	prospective prospective retrospective prospective prospective prospective retrospective retrospective prospective prospective prospective	supine NA prone NA NA NA NA NA NA NA NA	simultaneous simultaneous simultaneous simultaneous simultaneous simultaneous simultaneous simultaneous simultaneous simultaneous simultaneous
**PROGNOSIS**	Schiano, C., 2020 [56] Margolis, N.E., 2016 [57] Catalano, O.A., 2017 [58] Jena, A., 2017 [59] Jena, A., 2017 [60] Kong, E., 2018 [61] Incoronato, M., 2018 [62] Inglese, M., 2019 [63] Incoronato, M., 2019 [64] Morawitz, J., 2021 [65] Murakami, W., 2020 [66] Carmona-Bozo, J.C., 2021 [67]	40/217 (18.4%) 12/12 (100%) 21/21 (100%) 69/69 (100%) 98//98 (100%) 46/46 (100%) 50/50 (100%) 46/46 (100%) 77/155(49.7%) 56/56 (100%) 55/55 (100%) 32/32 (100%)	retrospective prospective retrospective prospective prospective prospective prospective prospective prospective prospective retrospective prospective	NA prone supine WB, prone B supine WB, prone B prone prone prone prone supine WB, prone B prone supine WB, prone B prone	simultaneous simultaneous simultaneous simultaneous simultaneous simultaneous simultaneous simultaneous simultaneous simultaneous simultaneous simultaneous
**RESPONSE**	Jena, A., 2017 [68] Wang, J., 2017 [69] Romeo, V., 2017 [70] Cho, N., 2018 [71] Andreassen, M.M.S., 2020 [72]	50/50 (100%) 14/14 (100%) 4/4 (100%) 26/26 (100%) 24/24 (100%)	prospective prospective prospective prospective prospective	supine WB, prone B prone NA supine WB, prone B NA	simultaneous simultaneous simultaneous simultaneous simultaneous

**Table 2:** Previous studies on PET/MRI evaluating the axillary status in breast cancer (NA = not available, WB = whole body PET/MRI, B = breast PET/MRI).

Authors	Total Number of Patients	Study Design	Patient Position	Type of Acquisition	Axillary Node Detection Sensitivity	Axillary Node Detection Specificity
Kirchner, J., 2018 [30]	38	prospective	supine WB, prone B	simultaneous	93%	95%
Botsikas, D., 2019 [32]	80	prospective	supine WB, prone B	sequential	0.85 (0.72–0.93)	0.89 (0.82–0.94)
Melsaether, A.N., 2016 [39]	51	prospective	supine	simultaneous	88–100% (CI 69, 97)	95% (CI 88, 98)
Taneja, S., 2014 [41]	36	retrospective	supine WB, prone B	simultaneous	60% on PET, 93.3% on MRI	91% on PET and MRI
Grueneisen, J., 2015 [42]	49	prospective	prone	simultaneous	78% (CI 52, 94)	90% (CI 74, 98)
Botsikas, D., 2016 [40]	58	retrospective	supine WB, prone B	sequential	79%	100%

**Table 3:** Ongoing studies evaluating the performance of PET/MRI in staging the axilla. (PST = Primary systemic Therapy, SNB = sentinel node biopsy, BC = breast cancer, TN = triple negative).

Study	Study ID	Study Site	Status	Study Design	Inclusion Criteria	PET/MRI Timing	Primary Outcome	Secondary Outcome
SNB vs. PET/MRI 1	ClinicalTrials.gov Identifier: NCT04826211	Milan, Italy	Recruiting	Single arm 110 participants	Candidates to PST Any T c/iN+	Before and after PST	Concordance rate between SNB and PET/MRI	Concordance rate between Axillary US vs. PET/MRI Correlation between PET/MRI parameters and prognosis
SNB vs. PET/MRI 2	ClinicalTrials.gov Identifier: NCT04829643	Milan, Italy	Recruiting	Single arm 247 participants	Candidates to upfront surgery with SNB cT < 3 cm cN0	Before surgery	Concordance rate between SNB and PET/MRI	Concordance rate between Axillary US vs. PET/MRI Correlation between PET/MRI parameters and prognosis
PET-MRI for Axillary Staging in Node Negative BC Patients	ClinicalTrials.gov Identifier: NCT03374826	Maastricht, The Netherlands	Recruiting	Single arm 125 participants	Candidates to upfront surgery with SNB cN0	Before surgery	PET/MRI accuracy in axillary staging	Accuracy of T2w MRI, DWI and Hybrid PET/MRI in axillary staging
Dedicated Breast PET/MRI in Evaluation of Extent of Disease in Women With Newly Diagnosed BC	ClinicalTrials.gov Identifier: NCT03510988	New York, USA	Suspended (due to COVID-19)	Single arm 147 participants	Women over age of 25 with newly diagnosed BC and for whom a breast MR has been ordered as standard of care	Prior to surgical and oncologic management	PET/MRI specificity by adding breast FDG PET to MR compared with breast MR alone for the diagnosis	Sensitivity in detection of axillary and internal mammary lymph node metastasis between the hybrid breast FDG PET/MRI vs. breast MRI alone
Initial Assessment of 18FDG-PET/MRI in Determining the Extent of Systemic Disease in BC Patients	ClinicalTrials.gov Identifier: NCT01672021	New York, USA	Active not recruiting	Single arm 80 participants	Patients with a history of BC undergoing PET/CT either for initial staging or for disease surveillance will perform PET/MRI	At diagnosis or onfollow-up	Number of metastatic lesions seen on PET/MRI compared with PET/CT	Patient stage as imaged by PET/MRI as compared with PET/CT
Whole-body staging in BC patients using combined MRI-PET	German Clinical Trials Register (DRKS; register number: DRKS00005410)	Dusseldorf, Essen, Germany	Recruiting complete, follow-up continuing	Single arm 199 participants	Newly diagnosed BC with one of the following features: T >= 2 TN BC of any size molecular high-risk (T1c + ki67% > 14%, Her2+, G3)	At diagnosis	Accuracy of combined whole-body 18F-FDG-MRI-PET for whole-body staging in BC	Comparison of combined whole-body 18F-FDG-MRI-PET and the diagnostic standard with regard to the diagnostic accuracy for whole-body tumor staging in breast cancer

**Table 4:** Italian SNB vs. PET/MRI trials: inclusion and exclusion criteria. (FNC = fine-needle cytology, PST = primary systemic therapy, BC = breast cancer, MRI = magnetic resonance imaging, Ax-US = axillary ultrasound, BCS = breast conserving surgery, SNB = sentinel node biopsy).

	SNB vs. PET/MRI 1	SNB vs. PET/MRI 2
**Inclusion criteria**	Any c/iT breast cancer Patients candidates to PST; Positive axillary nodes at diagnosis, confirmed by FNC. Patients with clear overt clinical and radiological nodal involvement might be enrolled as well without FNC.	cT ≤ 3 cm breast cancer cN0 (no palpable nodes) iN0 (no overt nodal involvement on Ax-US) Patients who are candidates to BCS or mastectomy plus SNB.
**Exclusion criteria**	inflammatory BC; pregnancy; distant metastases; no surgery after PST; claustrophobia; allergy to the MRI contrast agent, severe renal insufficiency.	inflammatory BC; pregnancy; distant metastases; claustrophobia; allergy to the MRI contrast agent; severe renal insufficiency.
